# Dependence of a Hydrogen Buffer Layer on the Properties of Top-Gate IGZO TFT

**DOI:** 10.3390/mi15060722

**Published:** 2024-05-29

**Authors:** Huixue Huang, Cong Peng, Meng Xu, Longlong Chen, Xifeng Li

**Affiliations:** 1 Shanghai Collaborative Innovation Center of Intelligent Sensing Chip Technology, Shanghai University, Shanghai 201800, China; hhx0519@163.com (H.H.); xumeng@shu.edu.cn (M.X.); 2 Key Laboratory of Advanced Display and System Applications of Ministry of Education, Shanghai University, Shanghai 200072, China; llchen@i.shu.edu.cn

**Keywords:** top-gate IGZO TFT, hydrogen-containing, buffer layer, technical computer-aided design simulation

## Abstract

In this paper, the effect of a buffer layer created using different hydrogen-containing ratios of reactive gas on the electrical properties of a top-gate In-Ga-Zn-O thin-film transistor was thoroughly investigated. The interface roughness between the buffer layer and active layer was characterized using atomic force microscopy and X-ray reflection. The results obtained using Fourier transform infrared spectroscopy show that the hydrogen content of the buffer layer increases with the increase in the hydrogen content of the reaction gas. With the increase in the hydrogen-containing materials in the reactive gas, field effect mobility and negative bias illumination stress stability improve nearly twofold. The reasons for these results are explained using technical computer-aided design simulations.

## 1. Introduction

In recent investigations of advanced semiconductors, amorphous oxide semiconductors, especially In-Ga-Zn-O (IGZO) ones, have been widely studied in relation to their ability to act as active layer materials for thin-film transistors (TFTs) due to their high field-effect mobility (*μ*), large-area uniformity (>Generation 8; 2200 mm × 2500 mm), low leakage current, low-temperature (<300 °C) fabrication process, and excellent transparency in the visible region [1,2,3,4,5,6]. At present, the structure of IGZO TFT can be roughly divided into a bottom gate and a top gate based on the active layer [3,5,6,7]. For the bottom-gate structure IGZO TFT, highly energetic particles generated in the deposition process used for a semiconductor channel (such as sputtering) are likely to cause damage to the dielectric. However, the dielectric in a top-gate IGZO TFT can serve as a gas permeation barrier [8]. In addition, a top-gate IGZO TFT is considered to be the most suitable structure for large high-resolution panel displays because it can provide better process controllability [7,9,10]. So, top-gate IGZO TFTs are receiving more and more attention from industry and academia [5,7,9,10,11]. In addition, IGZO films have become the most promising semiconductor materials in the flexible display field. Aluminum, stainless steel, and polyimide/polyethylene naphthalate have some issues when serving as the substrates of flexible displays, such as the electrical conductivity of aluminum and stainless steel and polyimide/polyethylene naphthalate’s poor adhesion to the device layer [12,13,14]. At the same time, there may be a stress mismatch between the substrate and the device layer. Therefore, to improve the applicability of top-gate IGZO TFTs, it is usually necessary to deposit a buffer layer on the substrate before fabricating TFT devices [12,13]. Typical buffer layers are composed of silicon dioxide (SiO_2_) or silicon nitride (Si_3_N_4_) deposited via plasma chemical vapor deposition (PECVD), and their preparation generally requires using some special gases as reaction sources, such as NH_3_, SiH_4_, and so on [3,7].

During the growth of the buffer layer, a large amount of hydrogen will be introduced into the film, and it is difficult to precisely control the hydrogen content within a reasonable range [7]. Some previous studies have shown that the hydrogen content of the buffer layer can be changed by changing how the buffer layers are stacked, the annealing temperature, or the type of substrate [2,13,14,15]. In addition, they have also put forward the idea that hydrogen can act as an impurity with a shallow donor state, and an appropriate hydrogen concentration in the buffer layer can improve the performance of devices such that they meet the requirements of display driving [7,9,13,14,15,16,17,18]. However, few studies have explored the preparation process for the precise control of the hydrogen content of the buffer layer, which affects the performance of a device. At the same time, there are hardly any reports that explain the effect of a buffer layer created using different hydrogen-containing ratios of reactive gas on the electrical properties of a top-gate IGZO TFT according to the density of states (DOSs) extracted using computer-aided design (TCAD) simulation. 

In this study, we adopted the method of adjusting the hydrogen-containing ratio of the reactive gas for the buffer layer to precisely control the performance of top-gate IGZO TFTs. The corresponding relationship between the hydrogen-containing ratio of the reactive gas and the hydrogen content in the buffer layer was ascertained via nondestructive Fourier transform infrared spectroscopy. The DOS of the channel layer was deduced based on TCAD simulation.

## 2. Experiment

**Figure 1:**
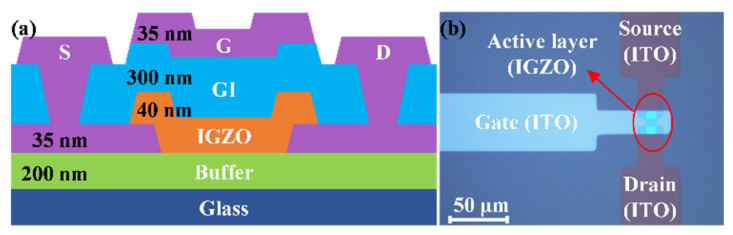
(**a**) The schematic diagram of top-gate IGZO TFTs. (**b**) Optical top view of top-gate IGZO TFT.

Staggered top-gate bottom-contact TFTs with IGZO channel layers were constructed on glass substrates. A schematic cross-sectional diagram and optical top view of the device are shown in Figure 1a,b, respectively. First, a 200 nm thick buffer layer was deposited on a 200 × 200 mm sheet of glass via PECVD, including SiO_2_ and Si_3_N_4_ in the process. Following this, a sheet of indium tin oxide (ITO) with a thickness of 35 nm and a sheet of IGZO with a thickness of 40 nm were sputtered via magnetically controlled sputtering as source/drain electrode (S/D) and active layers, respectively. Then, a SiO_2_ sheet with a thickness of 300 nm was successively deposited as a gate-insulating layer (GI) using PECVD. Finally, a 35 nm thick ITO thin film was sputtered again as a gate electrode (G) using magnetron sputtering. In addition, the patterning of each layer was achieved using a conventional lithography process. The width/length ratio (*W*/*L*) of all devices was 8/8 μm μm^−1^.

**Figure 2:**
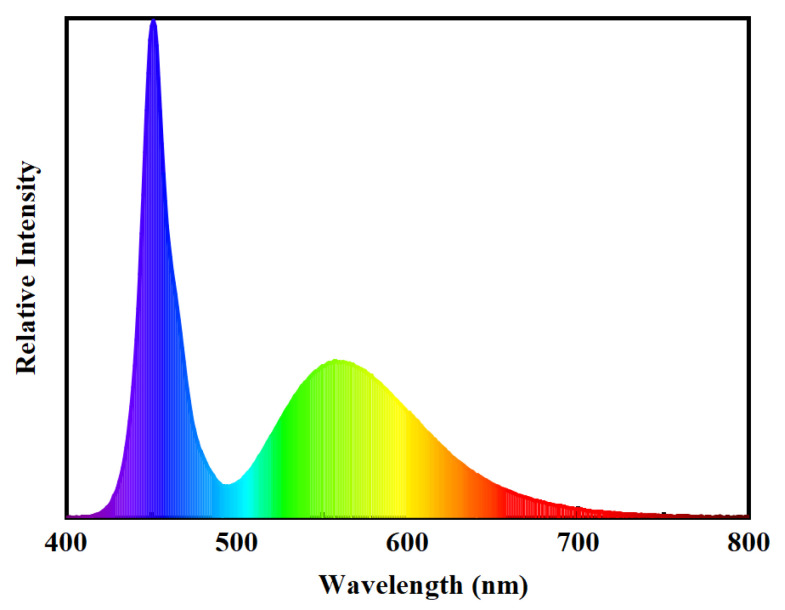
The emission spectra of the white LED backlight.

The surface morphology of the film was analyzed using an atomic force microscope (AFM, Bruker, Karlsruhe, Germany). The buffer/active layer interface roughness was analyzed using *X*-ray reflectivity (XRR, Smart lab, Tokyo, Japan). The hydrogen content of the buffer films was analyzed and calculated using Fourier transform infrared (FTIR, Nicolet 380, Thermo Fisher Scientific, Waltham, MA, USA) spectroscopy. The electrical performance of the devices was tested using a Keithley 4200 semiconductor, Tektronix, Beaverton, OR, USA) parameter analyzer. The transfer characteristics of all transfers were measured at a drain voltage of 10 V. The gate bias tests used were the negative bias stress (NBS) and negative bias illumination stress (NBIS) tests. The light source was a light-emitting diode (LED), whose light intensity was 10,000 lux, and the corresponding spectrum is shown in Figure 2. The threshold voltage (*V_TH_*) was determined from the x-axis intercept of the *I_DS_*^1/2^ versus *V_GS_* plot using the linear extrapolation method. The *μ* was calculated according to the following equation:

*μ* = *2L*·*I_DS_*/*W*·*C_i_*·(*V*_*GS*_ − *V*_*TH*_)^2^

*C_i_* is the gate capacitance per unit area.

## 3. Results and Discussion

### 3.1. Thin-Film Performance Analysis

To investigate the influence of the different hydrogen-containing ratios of the reactive gas in the buffer layer on the performance of thin films, the buffer layer films with a thickness of 200 nm were deposited on double-sided polished silicon wafers using PECVD (ULVAC, CME-200E) at 200 °C. As shown in Table 1, according to the hydrogen-containing ratios of the reactive gases in the developed buffer layer, the buffer layer is represented by normalized *N_H_*_0_, *N_H_*_3_, *N_H_*_28_, *N_H_*_93_, and *N_H_*_100_, respectively.

**Figure 3:**
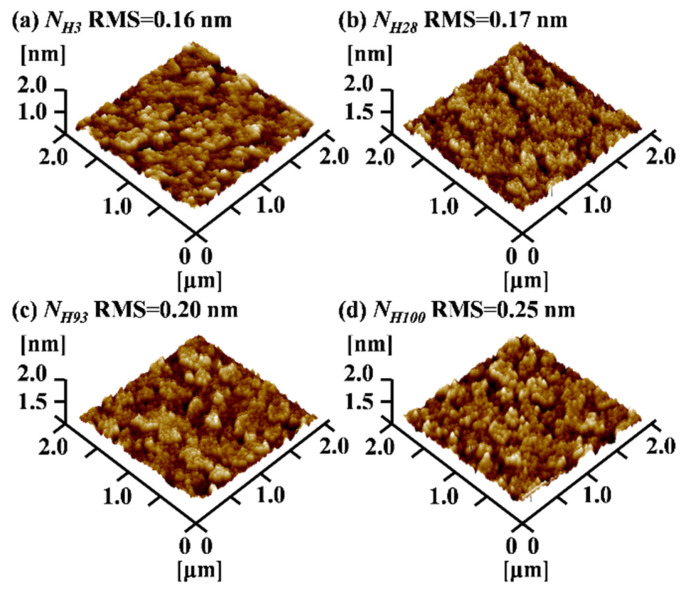
AFM 3D images of the morphology of the buffer layers: (**a**) *N_H_*_3_, (**b**) *N_H_*_28_, (**c**) *N_H_*_93_, and (**d**) *N_H_*_100_.

Figure 3a–d show the variation in the surface morphology of the buffer layer films with the hydrogen content of the growth gas obtained via atomic force microscopy (AFM). It can be seen that the surface root-mean-square roughness (RMS) increases from 0.16 nm to 0.25 nm as the hydrogen content increases from *N_H_*_3_ to *N_H_*_100_. The hydrogen concentration increased, which implies that some of the Si-O-Si bonding was replaced by H-terminated Si-OH and Si-H bonding on the surface of the buffer layer, enhancing the chemical reactivity of the silica surface and making it easier to oxidize or reduce the surface [19,20,21]. In addition, the increase in surface Si-H bonds makes it easier to form a loose porous structure, which affects the structure and morphology of the buffer layer thin-film surface, increasing surface roughness [21,22,23,24].

**Figure 4:**
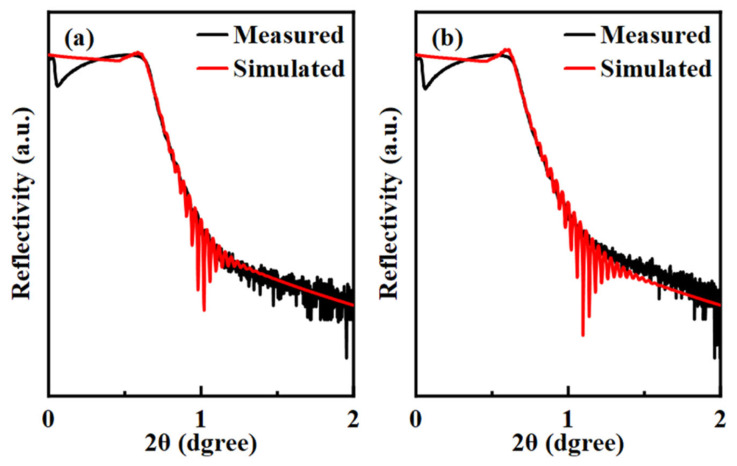
Measured (black solid line) and simulated (red solid lines) XRR spectra for the IGZO layers deposited on different buffer layers: (**a**) *N_H_*_3_/IGZO; (**b**) *N_H_*_100_/IGZO.

In particular, *N_H_*_3_ and *N_H_*_100_ buffer layer films were chosen as representative subjects based on the hydrogen-containing ratio of reactive gas. A 40 nm thick IGZO film was deposited on top of them (*N_H_*_3_/IGZO and *N_H_*_100_/IGZO), and then XRR was used to preliminarily determine the hydrogen-containing ratio of the reactive gas in the buffer layer on the interface between the buffer layer and the active layer. Figure 4 shows the measured (black solid line) and simulated (red solid line) XRR results for (a) *N_H_*_3_/IGZO and (b) *N_H_*_100_/IGZO. It can be seen that the simulation curve of the *N_H_*_3_/IGZO sample is smoother than that of *N_H_*_100_/IGZO, indicating that the interface of the former is smoother, and it produces less surface carrier scattering [25], which agrees sufficiently well with the AFM results. The IGZO densities of the *N_H_*_3_/IGZO and *N_H_*_100_/IGZO samples were 6.17 g/cm^−3^ and 6.15 g/cm^−3^, respectively, and this extremely small error was caused by system measurement or calculation errors, so the change in hydrogen content in the buffer layer did not cause the density change of the IGZO layer.

**Figure 5:**
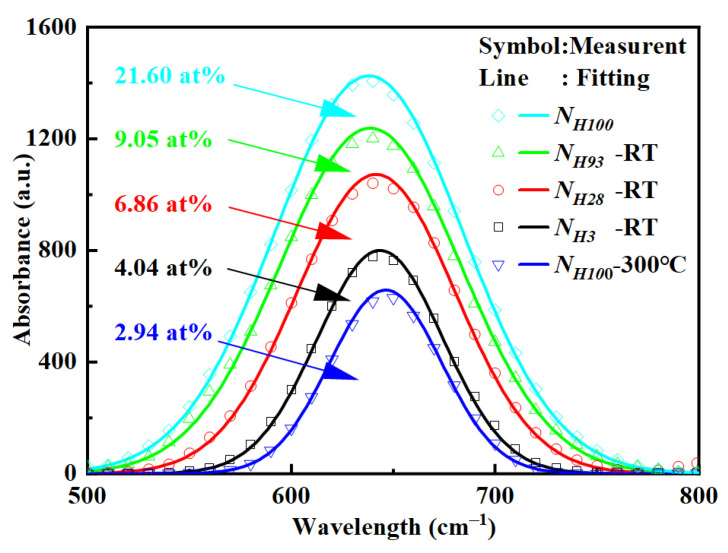
FTIR spectra at 640 cm^−1^ of the film deposited at different hydrogen−containing gas ratios of the buffer layers.

To clarify the corresponding relationship between the hydrogen-containing ratio of reactive gases for the buffer layer and the hydrogen content of the buffer layer film, the films deposited with different hydrogen-containing ratios of reactive gas were analyzed by FTIR. The absorption intensity of the FTIR absorption spectrum is positively correlated with the hydrogen content in the film, and the absorption peak near 640 cm^−1^ includes the wagging-rocking modes of Si-H wagging vibration [26]. Therefore, the hydrogen content is generally expressed by the intensity of the corresponding peak at 640 cm^−1^ [26,27]. The *C_H_* is determined using the following relationship: *C_H_* = *A_ω_*⋅*I(ω)*/*N*, *I(ω)* = ∫[*α(ω)*/*ω*]*dω*; here, *A*_640_ is the proportionality constant for this Si-H mode, with the value used for the films studied in this work being 1.6 × 10^19^ cm^−2^, and *N* is the atomic density of silicon atoms in c-Si, which is taken to be 5.0 × 10^22^ cm^−3^ [27]. Figure 5 depicts the absorption spectrum and Gaussian fitting results of the FTIR spectrum around 640 cm^−1^. As the ratio of hydrogen-containing reactive gases in the grown buffer layer increases from *N_H_*_0_ to *N_H_*_100_, the hydrogen content in the buffer layer film increases from 4.04 at% to 21.60 at%, as the higher the hydrogen content in the reaction gas, the higher the hydrogen content in the film. However, after annealing the buffer layer film of *N_H_*_100_, the hydrogen content decreased from 21.60 at% to 2.94 at%, which can be attributed to the diffusion of highly active hydrogen away from the buffer layer film [2,14,28,29].

### 3.2. The Influence of Hydrogen Content in the Buffer Layer

**Figure 6:**
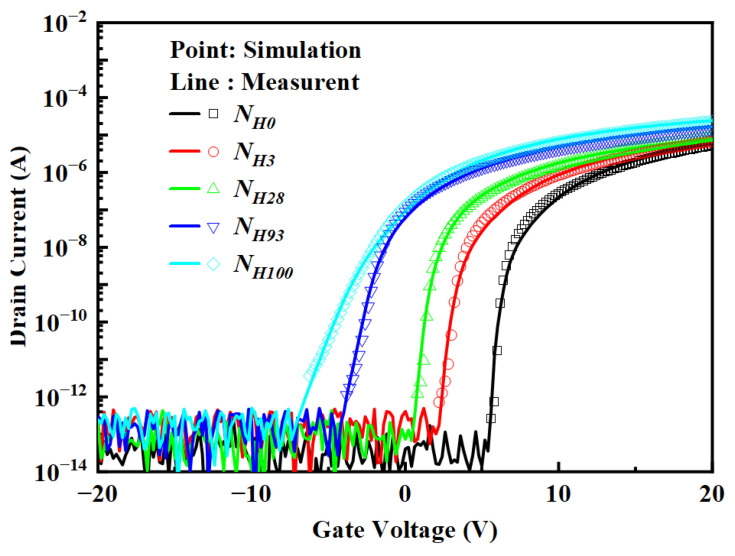
Comparison of experimental and simulated transfer characteristics.

Figure 6 shows the transfer characteristic curves of the top-gate IGZO TFT corresponding to different buffer hydrogen proportions after annealing. The device performance values are summarized in Table 2. As the ratio of hydrogen-containing reactive gases in the growth buffer layer increases from *N_H_*_0_ to *N_H_*_100_, the buffer capacitance per unit area (*C_i_*) increases from 16.83 to 31.45 nF/cm^2^, *μ* monotonically increases from 4.29 cm^2^V^−1^s^−1^ to 11.46 cm^2^/V·s, the on/off ratio for current (*I_on_/I_off_*) slowly increases from 1.14 × 10^8^ to 2.33 × 10^9^, the subthreshold swing (*SS*) increases from 0.16 V/dec to 0.86 V/dec, and *V_TH_* gradually shifts leftward from 7.78 V to −0.73 V.

**Figure 7:**
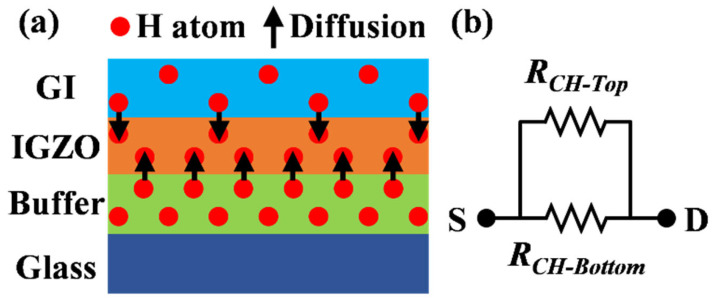
(**a**) Schematic diagram of hydrogen diffusion in adjacent layers of active layer. (**b**) Equivalent resistance model.

Since the hydrogen in the adjacent layers of IGZO will diffuse to the active layer, the hydrogen diffusion model shown in Figure 7a was established. The active layer was simplified to two equivalent resistances, the active layer resistance affected by the insulating layer is defined as *R_CH-Top_*, and the active layer resistance affected by the buffer layer is defined as *R_CH-Bottom_*, as shown in Figure 7b. Because the insulating layer growth process is the same for all top-gate IGZO TFTs, the hydrogen content diffused from the insulating layer into the IGZO can be considered to be approximately the same, and therefore the *R_CH-Top_* is the same for all the devices. Therefore, only the influence of hydrogen in the buffer layer on the performance of IGZO was considered. The hydrogen atoms in the buffer layer will diffuse into IGZO through the Buffer/IGZO interface and then combine with O^2−^ ions in the IGZO film to form hydroxyl groups, which are released electrons. As the hydrogen-containing ratio of reactive gases increases, the number of hydrogen atoms diffused into the IGZO also increases, so the electron concentration of the lower IGZO layer increases and the *R_CH-Bottom_* decreases, which is beneficial to the conduction of electrons. In general, the resistance between the source and drain electrodes is generated by the parallel resistance of the upper and lower layers of IGZO, so as the hydrogen content of the buffer layer increases, the total resistance between the source and drain electrodes will become smaller [30]. At the same time, the increase in the electron concentration of the entire channel will cause the device to be turned on in advance so that the *V_TH_* drifts to the left [13]. In addition, a higher carrier concentration also increases the mobility of IGZO [1]. With the increase in H concentration, donor and acceptor effects alternately play a leading role, as shown by the formation of the H_2_ molecule (-OH-H), which leads to the fluctuation of electrical parameters. In addition, excessive H can replace O in weak metal-oxygen bonds, which inhibits the bonding of interface metal-oxygen bonds and increases the number of interface defects, leading to the deterioration of *SS* [31,32].

### 3.3. 2D Numerical Simulation

A Silvaco ATLAS 2-D device simulator was used to investigate the differences in top-gate IGZO TFT devices with the different hydrogen-containing ratios of reactive gases for the buffer layer, particularly the difference in the DOS. Figure 6 shows the experimental and simulated transfer characteristics. Excellent agreement between the experiment and simulation was achieved. The sub-bandgap state nomenclature employed in Table 3 can be explained as follows. The acceptor-like tail states are defined by the peak density *N_TA_* and the Urbach energy (slope) *W_TA_*. The donor-like deep-level defect states are defined by the peak density *N_GD_*, the characteristic decay energy *W_GD_*, and the peak energy *E_GD_*. The density of fixed charges is represented by *QF*. The DOS and the key parameters of the defect model have the following relationship [33]:

DOS = *N_TA_*·exp[(*E* − *E_C_*)/*W_TA_*] + *N_GD_*·exp[−(*E* − *E_GD_*)^2^/*W_GD_*^2^]

Device performance was controlled by adjusting the hydrogen content in the buffer layer and controlling the diffusion of hydrogen-related impurities to adjust the hydrogen content in the IGZO. As the hydrogen-containing ratio of reactive gases increases from *N_H_*_0_ to *N_H_*_100_, *I_on_* increases from 5.79 × 10^−6^ A to 2.46 × 10^−5^ A, which can be attributed to the decrease in *N_TA_* from 1.57 × 10^20^ cm^−3^eV^−1^ to 6.00 × 10^19^ cm^−3^eV^−1^. The decrease in *N_TA_* means a decrease in acceptor-like tail states, which mainly capture free electrons transitioning to the conduction band so that there will be more free electrons transitioning to the conduction band at the same gate voltage [18,34]. In addition, the free electrons in the conduction band are conducted in the extended state, while the electron conduction in the band tail state consists of hopping conduction limited by traps, and its conductivity is much smaller than that of the extended state, so the electrons in the band tail state are conducted via hopping conduction limited by traps. The decrease in the concentration of trapped electrons will inevitably increase the effective mobility of electrons and increase *I_on_* [35,36]. While the hydrogen-containing ratio of reactive gas for the buffer layer increases from *N_H_*_0_ to *N_H_*_100_, the *SS* increases from 0.16 V/dec to 0.86 V/dec, which can be attributed to the increase in *N_GD_* from 3.00 × 10^17^ cm^−3^eV^−1^ to 4.30 × 10^17^ cm^−3^eV^−1^, and *QF* increases from 2.30 × 10^11^ cm^−2^ to 5.00 × 10^11^ cm^−2^. The change in *SS* is not substantially related to the change of acceptor-like tail states, mainly because acceptor-like tail states are closer to the conduction band, and their change does not affect the subthreshold region.

As the hydrogen-containing ratio of reactive gases increases from *N_H_*_0_ to *N_H_*_100_, the *V_TH_* shifts continuously to the left from 7.78 V to −0.73 V, which may be due to the increase in *N_GD_* from 3.00 × 10^17^ cm^−3^eV^−1^ to 4.30 × 10^17^ cm^−3^eV^−1^. An increase in *N_GD_* implies an increase in donor-like deep-level defects [18,37,38]. At the same voltage, the number of free electrons generated increases, and the number of electrons that can transfer to the conduction band also increases. This enables the device to turn on at a more negative gate voltage, so *V_TH_* shifts to the left [35,38,39]. The *E_GD_* decreased from 2.72 eV to 2.65 eV, indicating that the oxygen vacancy defect energy level shifted to the valence band, and the distance from the defect energy level to the bottom of the conduction band increased, which improved the NBIS stability of the device [20,40].

### 3.4. The Influence of Hydrogen Content on Stability

**Figure 8:**
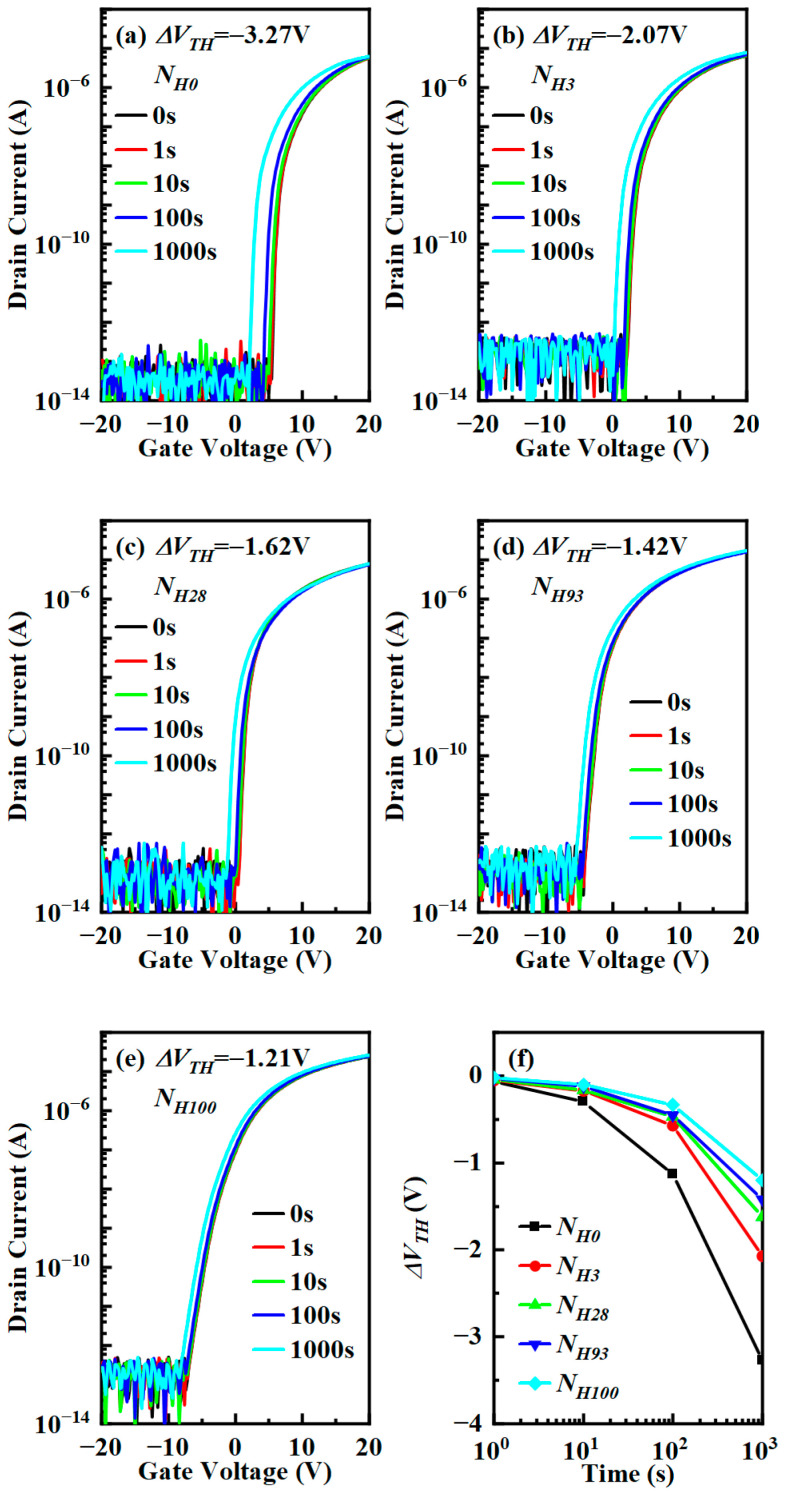
NBIS stability for top-gate IGZO TFT under different buffer hydrogen proportions: (**a**) *N_H_*_0_, (**b**) *N_H3_*, (**c**) *N_H_*_28_, (**d**) *N_H_*_93_, (**e**) *N_H_*_100_, and (**f**) Δ*V_TH_* as a function of stress time.

Figure 8 depicts the stability of top-gate IGZO TFTs with different hydrogen-containing ratios of reactive gases for the buffer layer under NBIS. With the increase in the hydrogen-containing ratio of reactive gases, Δ*V_TH_* reduced from −3.27 V to −1.21 V. Due to the presence of electrically neutral donor-like defect states introduced by oxygen-related defects in IGZO, the donor-like defect states will release electrons under NBIS and increase the carrier concentration in the channel, so the threshold voltage under NBIS will shift to the left. On the one hand, the V_O_ increases through the combination of hydrogen atoms with O^2−^ [41]. On the other hand, the hydrogen atom forms a substitutional impurity, forming a stable metal–hydrogen bond with metal ions, and the distance from the defect level to the bottom of the conduction band also increases, so the NBIS of the top-gate IGZO TFT improves [4,42,43].

## 4. Conclusions

In this paper, top-gate IGZO TFTs with different hydrogen proportions in the buffer layer were successfully fabricated, and the effect of hydrogen content on the stability of negative bias illumination stress was discussed. It has been found that the results of atomic force microscopy and *X*-ray reflection indicate that Si_3_N_4_ films with higher hydrogen content have greater surface and Si_3_N_4_/IGZO interface roughness, respectively. By optimizing the hydrogen content of the buffer layer, the field-effect mobility improved nearly threefold, reaching 11.46 cm^2^/V·s, while the NBIS stability was remarkably enhanced. TACD simulations further confirmed that deep donor-like and acceptor-like defects can be controlled by the hydrogen-containing ratio of reactive gases, consulting the reason for the remarkable performance of top-gate IGZO TFTs.

## Figures and Tables

**Table 1:** Values that were normalized to represent the hydrogen-containing ratios of reactive gas for the buffer layer.

Buffer	Reactive Gas	Reaction Gas Ratio	H%
w/o	Without	Without	*N_H_*_0_
SiO_2_	SiH_4_/N_2_O	4/700	*N_H_*_3_
		44/660	*N_H_*_28_
		144/560	*N_H_*_93_
Si_3_N_4_	SiH_4_/NH_3_/N_2_	40/154/510	*N_H_*_100_

**Table 2:** Electrical characteristics of top-gate IGZO TFTs with different hydrogen-containing gas ratios in the buffer layers.

Buffer	H%	*C_i_* (nF/cm^2^)	*μ* (cm^2^/V·s)	*I_on_*/*I_off_*	*SS* (V/dec)	*V_TH_* (V)
w/o	*N_H_*_0_	16.83 ± 0.03	4.29 ± 0.34	1.14 × 10^8^ ± 4.23 × 10^7^	0.16 ± 0.04	7.78 ± 0.33
SiO_2_	*N_H_*_3_	17.28 ± 0.04	5.74 ± 0.28	2.67 × 10^8^ ± 1.57 × 10^7^	0.21 ± 0.02	4.79 ± 0.26
	*N_H_*_28_	17.50 ± 0.04	7.85 ± 0.23	6.14 × 10^8^ ± 3.74 × 10^7^	0.23 ± 0.02	0.76 ± 0.30
	*N_H_*_93_	18.16 ± 0.02	8.24 ± 0.17	9.21 × 10^8^ ± 5.87 × 10^7^	0.34 ± 0.03	−0.56 ± 0.15
Si_3_N_4_	*N_H_*_100_	31.45 ± 0.05	11.46 ± 0.15	2.33 × 10^9^ ± 3.66 × 10^8^	0.86 ± 0.07	−0.73 ± 0.21

**Table 3:** Densities of key defect model parameters for top-gate IGZO TFTs fitted according to different hydrogen-containing gas ratios of the buffer layers.

H%	*N_TA_*	*W_TA_*	*N_GD_*	*W_GD_*	*E_GD_*	*QF*
	cm^−3^eV^−1^	eV	cm^−3^eV^−1^	eV	eV	cm^−2^
*N_H_*_0_	1.57 × 10^20^	0.032	3.00 × 10^17^	0.12	2.72	2.30 × 10^11^
*N_H_*_3_	1.55 × 10^20^	0.032	3.20 × 10^17^	0.12	2.70	2.60 × 10^11^
*N_H_*_28_	1.40 × 10^20^	0.032	3.50 × 10^17^	0.12	2.68	3.00 × 10^11^
*N_H_*_93_	1.00 × 10^20^	0.032	4.00 × 10^17^	0.12	2.67	4.50 × 10^11^
*N_H_*_100_	6.00 × 10^19^	0.032	4.30 × 10^17^	0.12	2.65	5.00 × 10^11^

## Data Availability

The data and contributions presented in the study are included in this article. Further inquiries can be directed to the corresponding author.
